# Longitudinal whole blood transcriptomic analysis characterizes neutrophil activation and interferon signaling in moderate and severe COVID-19

**DOI:** 10.1038/s41598-023-37606-y

**Published:** 2023-06-26

**Authors:** Christian Prebensen, Yohan Lefol, Peder L. Myhre, Torben Lüders, Christine Jonassen, Anita Blomfeldt, Torbjørn Omland, Hilde Nilsen, Jan-Erik Berdal

**Affiliations:** 1 Department of Infectious Diseases, Oslo University Hospital, Kirkeveien 166, 0450 Oslo, Norway; 2 Institute of Clinical Medicine, University of Oslo, Oslo, Norway; 3 Department of Microbiology, University of Oslo, Oslo, Norway; 4 Department of Cardiology, Akershus University Hospital, Lørenskog, Norway; 5 Department of Clinical Molecular Biology, Akershus University Hospital, Lørenskog, Norway; 6 Center for Laboratory Medicine, Østfold Hospital Trust, Grålum, Norway; 7 Department of Microbiology and Infection Control, Akershus University Hospital, Lørenskog, Norway; 8 Department of Infectious Diseases, Akershus University Hospital, Lørenskog, Norway

**Keywords:** Inflammation, Innate immune cells, Infectious diseases, Viral infection, Translational research, Viral infection, Molecular medicine

## Abstract

A maladaptive inflammatory response has been implicated in the pathogenesis of severe COVID-19. This study aimed to characterize the temporal dynamics of this response and investigate whether severe disease is associated with distinct gene expression patterns. We performed microarray analysis of serial whole blood RNA samples from 17 patients with severe COVID-19, 15 patients with moderate disease and 11 healthy controls. All study subjects were unvaccinated. We assessed whole blood gene expression patterns by differential gene expression analysis, gene set enrichment, two clustering methods and estimated relative leukocyte abundance using CIBERSORT. Neutrophils, platelets, cytokine signaling, and the coagulation system were activated in COVID-19, and this broad immune activation was more pronounced in severe vs. moderate disease. We observed two different trajectories of neutrophil-associated genes, indicating the emergence of a more immature neutrophil phenotype over time. Interferon-associated genes were strongly enriched in early COVID-19 before falling markedly, with modest severity-associated differences in trajectory. In conclusion, COVID-19 necessitating hospitalization is associated with a broad inflammatory response, which is more pronounced in severe disease. Our data suggest a progressively more immature circulating neutrophil phenotype over time. Interferon signaling is enriched in COVID-19 but does not seem to drive severe disease.

## Introduction

Although SARS-CoV-2 infection leads to mild disease in a majority of patients, progression to severe respiratory failure occurs in a substantial proportion, particularly in the absence of protective vaccine responses. A large body of evidence supports the pivotal role of a maladaptive inflammatory response in the pathogenesis of severe COVID-19, but it remains unclear to what extent the immune response contributing to severe disease is distinct from that of mild and moderate disease. Studies applying both RNA sequencing and high-dimensional cytometry have identified dysregulated neutrophil and monocyte populations in critically ill patients^1–8^, and aberrant or delayed interferon signaling has also been implicated in severe disease^9–14^. Anti-inflammatory therapies have shown the potential to reduce mortality in patients hospitalized with severe COVID-19^15,16^, but a more thorough understanding of the nature and kinetics of the immune response to SARS-CoV-2 in patients with severe vs. moderate disease may help to optimize patient selection and the timing of immunomodulatory treatment. The aim of this study was therefore to further investigate the dynamic host response profile of nonvaccinated patients hospitalized with severe and moderate COVID-19, using microarray and protein biomarker analysis of serial peripheral blood samples.

## Methods

The study was approved by the Regional Committees for Medical Research Ethics South-East Norway (reference number 2020_39) and was performed in accordance with the Declaration of Helsinki. Written informed consent was granted by study participants or legal guardian/next-of-kin.

### Patient inclusion

Between March and May 2020, 135 patients admitted to Akershus University Hospital with COVID-19 confirmed by SARS-CoV-2 RT-PCR were prospectively recruited to the Coronavirus Disease Mechanisms (COVID MECH) observational cohort study. Thirty-six patients (27%) were admitted to the ICU and eight (6%) died. This substudy included: 17 patients with severe disease according to the WHO ordinal severity scale^17^ and all requiring ICU admission and invasive mechanical ventilation, and 15 patients with moderate disease receiving supplemental O_2_ on regular medical wards. Patients were selected based on the availability of sequential whole blood RNA samples, relatively uniform clinical trajectories and time from symptom onset to baseline sampling between five and 14 days. No patients received corticosteroids, other potent anti-inflammatory therapy, or remdesivir. RNA samples from 11 healthy volunteers matched to patients by age and gender served as controls. Controls had no current or recent symptoms of infection, nor were they taking any immunosuppressive medication at the time of sampling. Patients and controls were unvaccinated, as the study predated available vaccines against SARS-CoV-2.

### Sampling

Samples, comprising EDTA plasma, serum and whole blood in PAXgene RNA tubes, were collected at study baseline (T1), after 1–4 days (mean 2.3 days, annotated as T2) and 5–11 days (mean 7.9 days, annotated as T3), where possible.

### Biochemistry

Routine biochemistry parameters including leukocyte differential counts were analyzed in the clinical laboratory. CRP, IL-6, cardiac troponin T, NT-proBNP and GDF-15 were analyzed in serum by the electrochemiluminescence immunoassay Elecsys on the Cobas e801 platform (Roche Diagnostics, Rotkreuz, Switzerland). ST2 was measured in serum using the Presage ST2 assay (Critical Diagnostics, San Diego, California, USA.

### Plasma SARS-CoV-2 RNA

Total nucleic acids were extracted from 200 µl of plasma using the MagNA Pure 96 system (Roche, Penzberg, Germany) and eluted in 50 µl. SARS-CoV-2 RNA was detected from 5 µL eluate in a 25 µL reaction mix on a QuantStudio™ 7 PCR system (Thermofisher Scientific, Waltham, MA, USA) in duplicate reactions, according to the method published by Corman et al*.*^18^. SARS-CoV-2 RNA quantitation was calculated using a serial dilution of a synthetic Wuhan coronavirus 2019 E gene RNA control, provided by the European Virus Archive Global (EVAg). The limit of detection (LoD) and limit of quantitation (LoQ) of the assay was 2.29 and 2.70 log_10_ RNA copies/mL plasma, respectively.

### Serology

Total antibodies against SARS-CoV-2 nucleocapsid (NP) were quantified using the Elecsys Anti-SARS-CoV-2 test on the cobas e801 module (Roche, Penzberg, Germany). IgG antibodies directed against subunits S1 and S2 of the SARS-CoV-2 spike protein were quantified using the LIAISON SARS-CoV-2 S1/S2 IgG assay and the Liaison XL chemiluminescence analyzer (DiaSorin, Saluggia, Italy).

### RNA extraction and microarray analysis

RNA was extracted from PAXgene tubes using the PAXgene Blood miRNA Kit (Qiagen, Hilden, Germany). 25 ng RNA was amplified and labelled with Cy3 using the Low Input Quick Amp Labeling Kit, one-color (Agilent, CA, USA), and the labelled cRNA was purified using the Qiagen RNeasy Mini Kit. Amplification and labelling efficiency were controlled on a NanoDrop ND-1000 spectrophotometer (Thermo Fisher, MA, USA). cRNA quality was assessed by measuring the RNA absorbance ratio 260 nm/280 nm. The specific activity of labeled cRNA (pmol Cy3 per ug cRNA) was defined to be 6 or above. 19 $\upmu$L of cRNA per sample was fragmented and applied to Agilent SurePrint G3 Human Gene Expression v3 8 × 60 k Microarrays. After hybridization for 17 h at 65 °C the microarray slides were washed, scanned with the Agilent Microarray Scanner and the data extracted using Agilent Feature Extraction Software.

### Bioinformatic workflow

Microarray data processing, normalization, principal components analysis (PCA) and differential gene expression was performed using limma^19^ version 3.48.1*.* Differentially expressed genes (DEGs) were defined by a p-value threshold of 0.05 and an absolute log fold change threshold of 1. Gene set enrichment analysis (GSEA) was performed using clusterProfiler^20^ version 4.0.0, with p-value and false discovery rate cutoff of 0.05. Semantic similarity was calculated using the ‘Wang’ method^21^ and plotting was performed using the enrichplot package, version 1.12.1. Weighted correlation network analysis (WGCNA) was performed using the WGCNA package^22^, version 1.70.3. All non-binary clinical values were log_10_-transformed for WGCNA. The power value, calculated using the ‘plot_power’ function of WGCNA, was found to be 18. Over-representation analysis was performed for each module using g:Profiler^23^, version e109_eg56_p17_1d3191d. CIBERSORT analysis^24^ was performed with the LM22 reference matrix.

### Time series analysis

Time series analyses were performed using an in-house pipeline^25^. In brief, differential gene expression analysis was performed using limma for each conditional and temporal comparison. Significant DEGs were pooled and clustered using the clusterGenomics^26^ package, version 1.0. Functional enrichment of each cluster was obtained using g:Profiler^23^. Mean expression of each cluster in healthy controls was determined by a permutation approach where all healthy samples were assigned to every possible timepoint.

### Statistics

Clinical data are presented as mean (+/− SD) or median (Q1, Q3) as appropriate. Group differences in clinical, biomarker and CIBERSORT data were analyzed by T-tests, Mann–Whitney U tests and Fisher’s exact tests.

Analyses were performed using R version 4.3.0 and Stata SE version 16.

## Results

Baseline clinical and plasma biomarker data is presented in Table 1 (data at all timepoints can be found in Supplementary File 1). There was no difference in age, sex, BMI or days from symptom onset to study baseline between patients with severe and moderate COVID-19. Patients with severe disease had higher circulating concentrations of CRP, IL-6, GDF-15, ST-2 and D-dimer, as well as higher SARS-CoV-2 RNAemia. Median neutrophil/lymphocyte ratio was higher in patients with severe disease, although the difference was not statistically significant.

**Table 1:** Baseline clinical and biomarker data.

	Severe	Moderate	p-value (severe vs. moderate)	Healthy controls	p-value (all COVID-19 vs. controls
N	17	15		11	
Age, mean (SD)	62.1 (8.5)	54.7 (15.4)	0.10	59.7 (6.1)	0.78
Male sex, #	11 (65%)	9 (60%)	0.78	5 (45%)	0.32
In-hospital mortality, #	3 (18%)	0 (0%)	0.09	–	
BMI, mean (SD)	29.3 (7.9)	29.9 (9.1)	0.84	–	
Days of symptoms, mean (SD)	9.6 (2.2)	9.2 (2.8)	0.67	–	
Worst WHO score (SD)	7.9 (1.2)	4.8 (0.4)	< 0.001	–	
SAPS-II score, mean (SD)	37.3 (6.9)				
Duration of ICU treatment, median days (Q1, Q3)	16 (12, 48)				
Cardiovascular disease, #	2 (12%)	1 (7%)	0.62	–	
Diabetes mellitus, #	3 (18%)	2 (13%)	0.74	–	
RNAemia, median copies/mL (Q1, Q3)	1554 (348, 6656)	0 (0, 845)	0.005		
Neutrophil count, median cells/ $\upmu$L (Q1, Q3)	7.5 (5.9, 10.7)	4.4 (3.1, 6.1)	0.05		
Neutrophil–lymphocyte ratio, median (Q1, Q3)	8.1 (3.5, 11. 9)	4 (2.8, 5.5)	0.17		
CRP, median mg/L (Q1, Q3)	190 (90, 260)	80 (37, 170)	0.05	–	
Ferritin, median ug/L (Q1, Q3)	509 (330, 1205)	823 (267, 1183)	0.90	–	
IL6, median pg/mL (Q1, Q3)	162 (75, 230)	40 (13, 51)	< 0.001	–	
PCT, median $\upmu$g/L (Q1, Q3)	0.24 (0.11, 1.1)	0.11 (0.09, 0.21)	0.07	–	
Troponin T, median ng/L (Q1, Q3)	12 (7, 19)	8 (4, 15)	0.14		
NT-proBNP, median ng/L (Q1, Q3)	177 (83, 480)	101 (23, 325)	0.32		
GDF-15 median pg/mL (Q1, Q3)	3940 (3096, 4533)	2080 (1226, 3191)	0.004	–	
ST2, median mg/mL (Q1, Q3)	73.8 (54.8, 92.3)	40.4 (30.8, 47.0)	0.001	-	
D-dimer, median mg/L (Q1, Q3)	0.8 (0.4, 1.6)	0.4 (0.3, 0.8)	0.05	–	

Abbreviations: SD: Standard deviation. Q1, Q3: First and third quartile, respectively.

P-values derived from T-tests, Mann–Whitney U tests and Fisher’s exact tests as appropriate.

### Broad inflammatory response in COVID-19 patients vs. healthy controls

**Figure 1:**
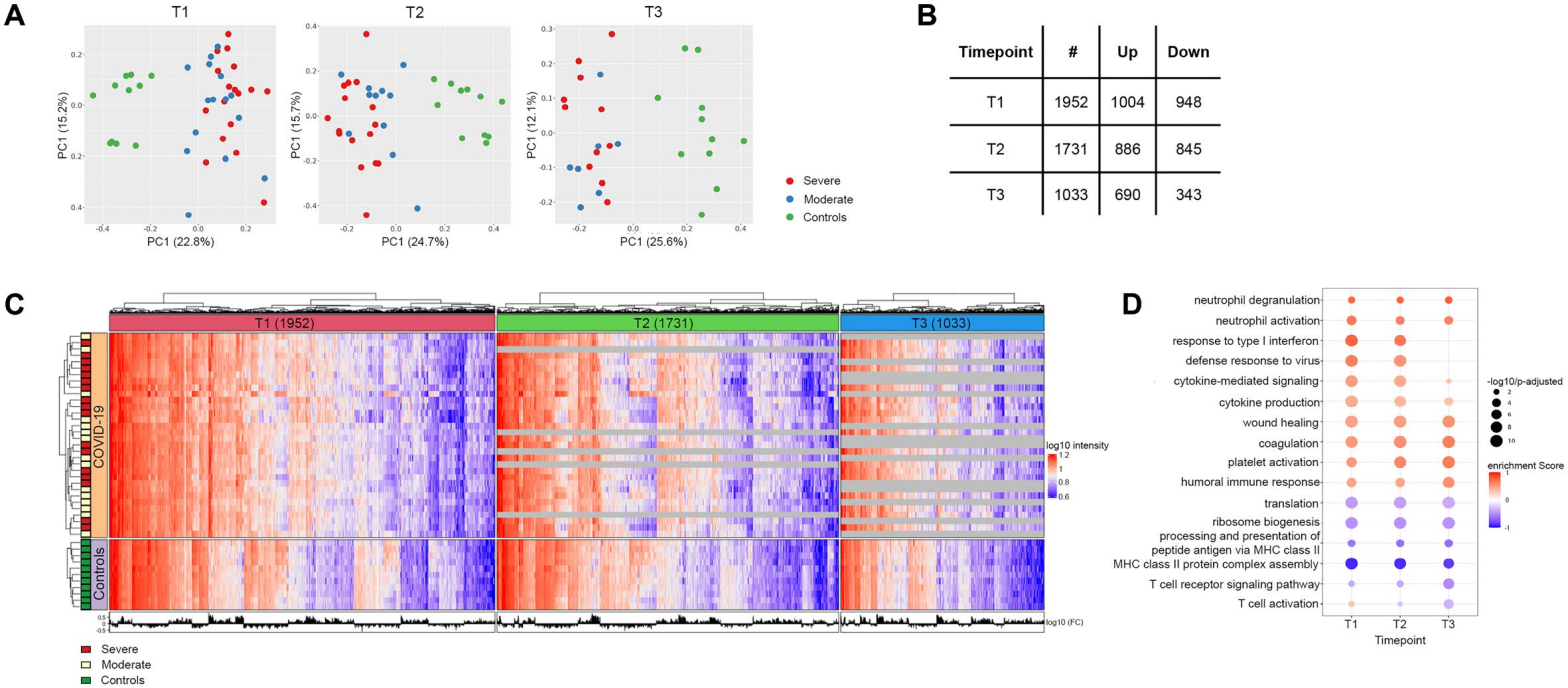
COVID-19 patients vs. controls. (**A**) Principal component analysis of gene expression data from all samples at three timepoints. Severe COVID-19 in red, moderate COVID-19 in blue, healthy controls in green. Percentage of explained variance in parentheses. (**B**) Differentially expressed genes (DEGs) per sampling timepoint. (**C**) Heat map with significant DEGs on the X axis and study participants on the Y axis. Patient group indicated on the left. (**D**) Enriched immune-related gene ontology (GO) terms in COVID-19 patients vs. controls. Positively enriched terms in COVID-19 in red and negatively enriched terms in blue. Dot size indicates the significance of the GO term enrichment based on the negatively transformed adjusted p-value.

Data from 88/92 arrays passed quality control and were analyzed further. Initial data exploration was performed by Principal Component Analysis (PCA), demonstrating clear separation between patients and healthy controls in PC1, but no separation between patients with severe and moderate disease in PC1 or 2 (Fig. 1A).

Comparing all COVID-19 patients to healthy controls, we identified 1952, 1731 and 1033 DEGs at T1, T2 and T3, respectively (Fig. 1B, Supplementary File 2). Hierarchical clustering did not suggest that the patients separated in distinct subgroups, although some variation could be seen (Fig. 1C). Applying Gene Set Enrichment Analysis (GSEA), multiple immune-related Gene Ontology (GO) terms were significantly enriched in patients (Fig. 1D).

At all timepoints, GSEA suggested a pronounced activation of neutrophils, platelets and coagulation in COVID-19, but also enrichment of the Wound healing GO term (including genes *IGF1* and *SDC1*), suggesting ongoing immune activation by Danger-Associated Molecular Patterns (DAMPs). Cytokine production and signaling (including *FFAR3* and *IL27*) was significantly increased in COVID-19, most markedly at T1 and T2. On the other hand, the Humoral response GO term, reflecting both complement (*SERPING1, C1QB*) and B cell activation (*JCHAIN*, *POU2AF1*), was progressively more enriched in COVID-19 patients from T1 through T3 (Fig. 1D, Supplementary File 3).

Antiviral response GO terms, including type 1 and type 2 interferon (IFN) signaling, were significantly enriched in COVID-19 at T1 and T2. However, *IFN-α* was the only consistently detected IFN transcript, and *IFN-α* expression was not significantly elevated in patients with COVID-19 compared to healthy controls.

While the T cell activation GO term was modestly enriched in COVID-19 at T1, we observed negative regulation of T cell function and reduced T cell receptor signaling in this group at all timepoints (Supplementary File 3). The expression of numerous class II HLA genes (Supplementary File 2) was reduced in COVID-19 at all timepoints, and the GO terms RNA processing, translation and ribosome biogenesis were consistently underrepresented.

### Severe COVID-19 is associated with more pronounced inflammation

**Figure 2:**
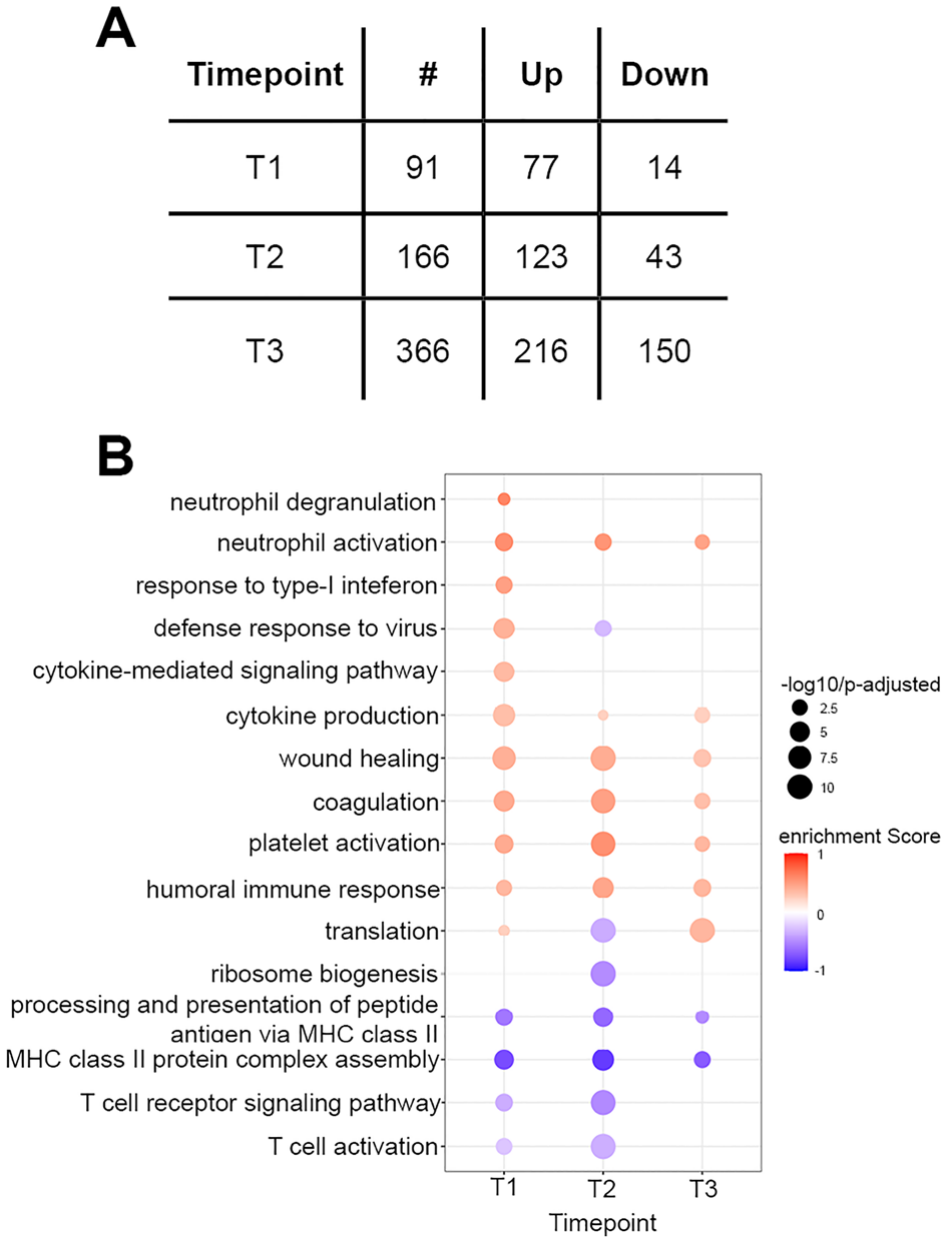
Severe vs. moderate COVID-19. (**A**) DEGs in severe vs. moderate COVID-19. (**B**) Enriched immune-related GO terms in severe vs. moderate patients. Positively enriched terms in severe disease in red and negatively enriched terms in blue. Dot size indicates the significance of the GO term enrichment based on the negatively transformed adjusted p-value.

Comparing patients with severe and moderate COVID-19 we identified 91, 166 and 366 DEGs at T1, T2 and T3, respectively (Fig. 2A, Supplementary File 2).

Similar to the comparison of disease vs. controls (Fig. 1D), GSEA showed enrichment of neutrophil activation, cytokine production, platelet activation, coagulation, wound healing and humoral immune response GO terms at all timepoints in severely ill patients (Fig. 2B). By contrast, our data suggested that class II HLA antigen presentation was most downregulated in patients with severe disease, with GO terms underrepresented at all timepoints. Similarly, T cell activation and T cell receptor signaling was reduced in severe disease at T1 and T2.

Interestingly, IFN signaling GO terms were enriched at T1 in patients with severe disease but the Defense response to virus GO term was underrepresented at T2 compared to patients with moderate disease.

Separate comparisons of patients with severe and moderate COVID-19 to healthy controls demonstrated highly overlapping DEGs and GO term enrichment in the two groups (Supplementary File 2, Supplementary Fig. 2).

### Time series analysis demonstrates divergent neutrophil gene trajectories

**Figure 3:**
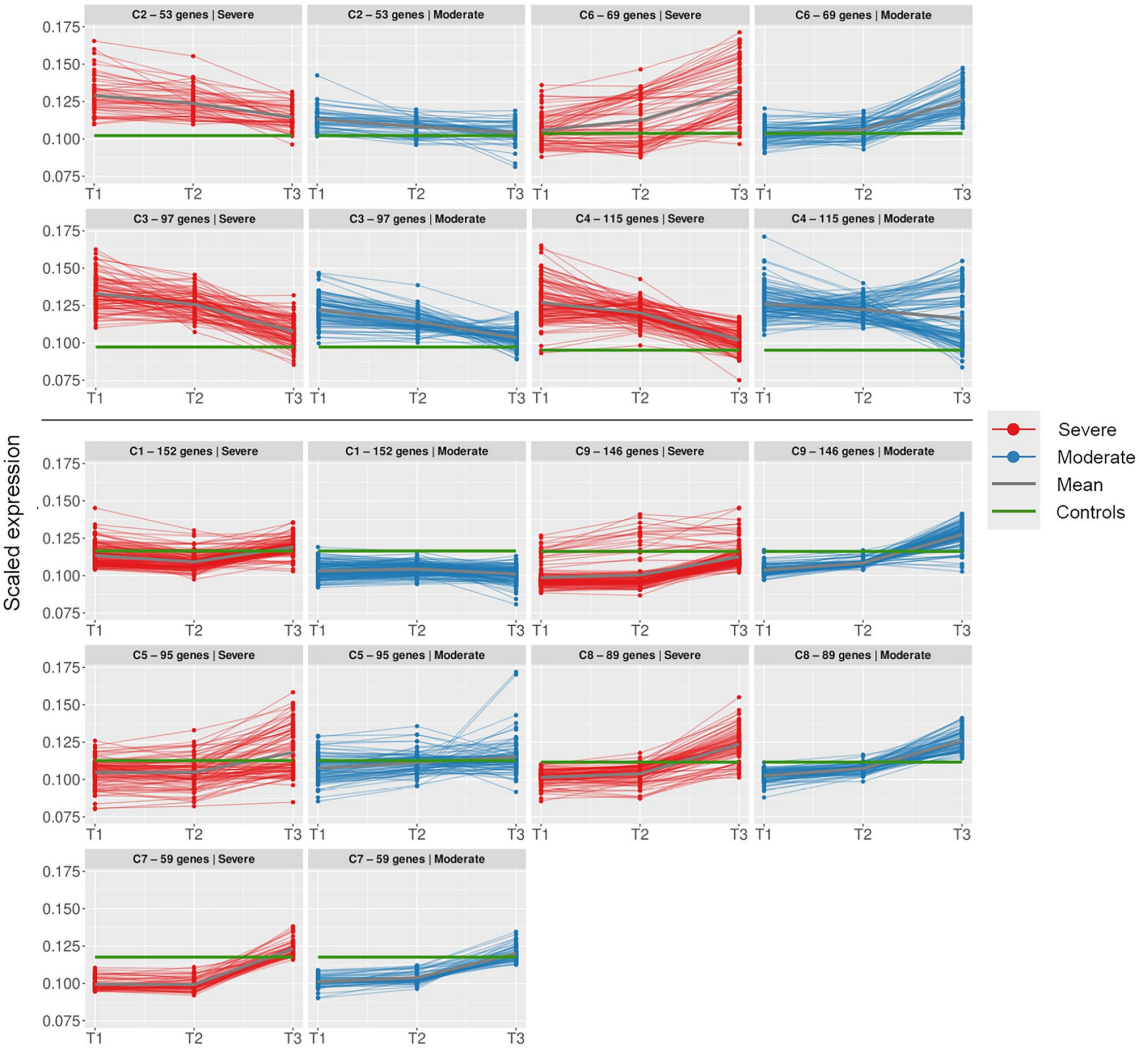
Time series. Trajectories of gene clusters in time series analysis. Upper panel shows clusters of particular interest: C2 and C6 contain neutrophil-related genes, C3 and C4 contain IFN-related genes. Scaled gene expression in each cluster is coloured by patient group: severe in red, moderate in blue. Mean expression in patients is indicated by a thick grey line, mean expression in healthy controls by a green line. Timepoints indicated on the X-axis.

To further explore the dynamics of the COVID-19 immune response, we performed a time series analysis, identifying nine clusters of genes with similar expression patterns over time (Fig. 3, Supplementary Fig. 3). Overrepresentation analysis identified four clusters with highly significant enrichment of GO terms, suggesting functional relevance (Supplementary File 4). These clusters also exhibited distinct temporal trajectories.

Clusters 2 and 6 were both enriched for neutrophil-associated genes, and gene expression in both clusters was higher in patients with severe disease, but with inversed trajectories. Cluster 2, containing neutrophil activation genes *CD177*, *MCEMP1* and *RNASE2* (Supplementary File 5) was highly expressed in early disease and decreased over time. Cluster 6, containing a larger number of neutrophil-associated genes, including *RETN, ARG1, ELANE, MMP8, BPI, AZU1, HP* and *LTF*, displayed increased expression over time in both patient groups.

Interferon signaling and antiviral immune response genes (Supplementary File 5) were enriched in clusters 3 and 4, which both displayed downward trajectories over time in patients, irrespective of severity. Cluster 3 included interferon-associated genes *IFI27, IFIT2* and *PARP9/14*, cytokine signaling genes *FFAR3*, *TNFAIP6*, *SOCS3* and *IL1R1/2* and immune checkpoint molecule *CD274* (PD-L1), and mean expression was higher in severe patients at T1 and T2. Subclustering to further delineate the trajectory of the IFN-associated genes demonstrated that expression was higher in severe patients only at T1, with comparable expression in later disease, consistent with GSEA (Supplementary File 4, Supplementary Fig. 4). Cluster 4, on the other hand, contained interferon-associated genes such as *IFIT1/3/5*, *ISG15*, *OAS1/2/3/L*, *APOBEC3B*, *PARP10* and *STAT1* as well as a number of immunoglobulin transcripts. Whereas mean expression in cluster 4 was comparable in patients with severe and moderate disease, gene trajectories were somewhat heterogeneous. Subclustering revealed that while the trajectory of interferon-associated genes was consistent with cluster 4 overall, ie. diminishing comparably over time, the expression of immunoglobulin genes increased throughout the disease course in patients with moderate disease, but diminished from T2 to T3 in severely ill patients (Supplementary File 4, Supplementary Fig. 4). SARS-CoV-2-specific antibodies (total anti-NP and anti-Spike S1/S2 IgG) in serum increased similarly in both patient groups from T1 to T3 (Supplementary Fig. 5).

### Severity-associated WGCNA modules overlap with time series gene clusters

To identify gene sets correlating with clinical variables associated with each sample we performed weighted gene co-expression network analysis (WGCNA, Supplementary Figs. 6 and 7). We focused on modules with significant positive or negative associations with WHO severity score and characterized them by over-representation analysis.

**Figure 4:**
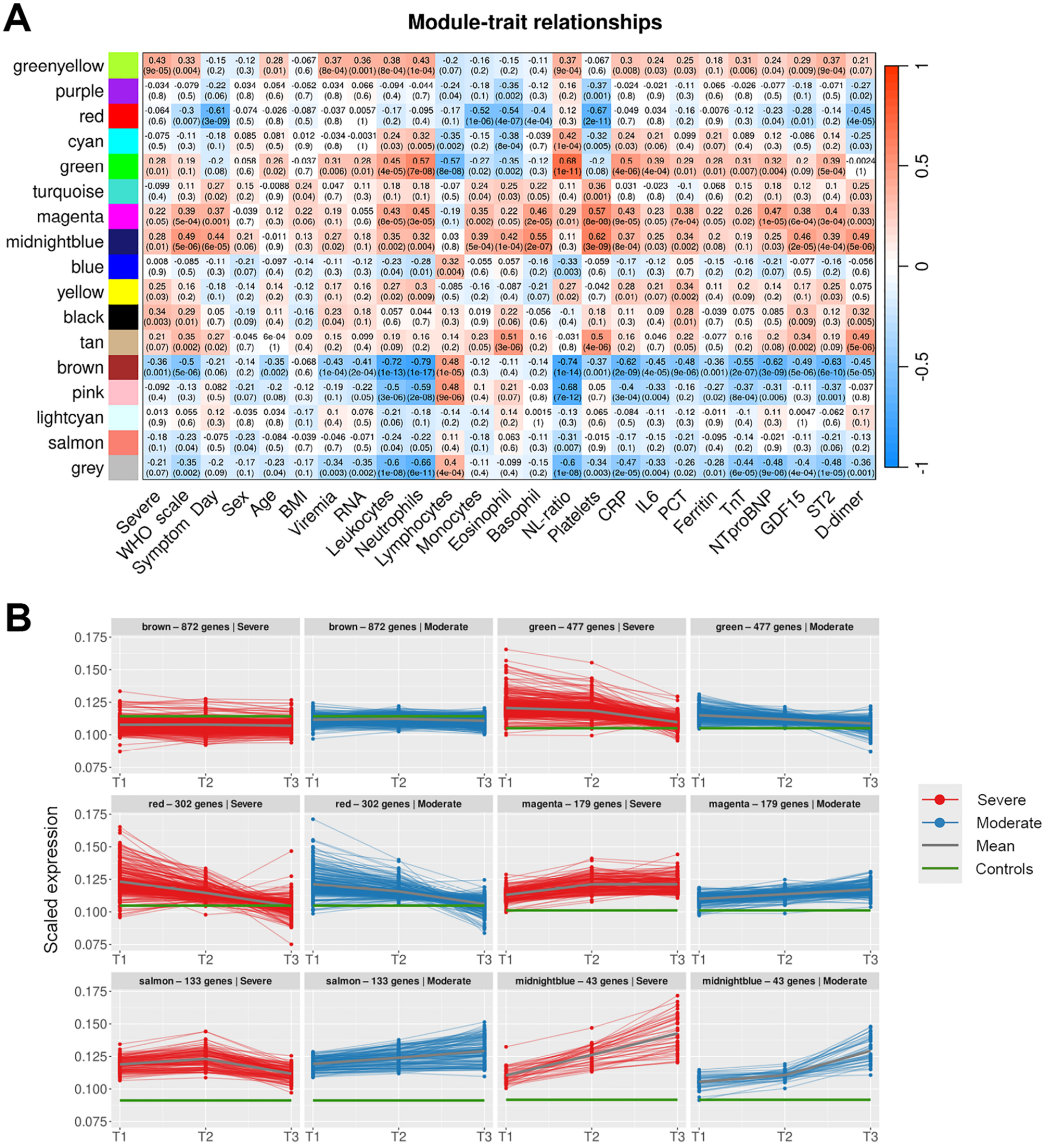
WGCNA. (**A**) Correlations between WGCNA gene modules and clinical traits. For each correlation, the correlation coefficient and p-value (in parentheses) are provided. Positive correlations are in red, negative in blue. (**B**) Trajectories of genes in WGCNA modules associated with WHO severity score. Remaining trajectories are presented in Supplementary Fig. 7. Scaled gene expression in each module is coloured by patient group: severe in red, moderate in blue. Mean expression in patients is indicated by a thick grey line, mean expression in healthy controls by a green line.

The MidnightBlue module correlated strongly with WHO disease severity, leukocyte and platelet counts, symptom day and concentrations of GDF-15 and D-dimer (Fig. 4A). This module consisted of neutrophil-associated GO terms (Supplementary File 6) and genes *LTF, LCN2, ELANE, CEACAM8, BPI, MMP8, AZU1, DEFA3* and *DEFA4* (Supplementary File 7), and markedly overlapped with time series cluster 6, which similarly increased over time (Fig. 4B).

The Magenta module also correlated strongly with WHO severity score, leukocyte counts and concentrations of GDF-15, as well as concentrations of CRP, NT-proBNP, procalcitonin and ST-2. Comprising platelet activation, coagulation and wound healing GO terms and platelet-associated genes such as *MYL9*, *ITGB3*, *GP1BB*, *TBXA2R* and *GP6*, expression of the Magenta module increased modestly over time and was consistently higher in patients with severe COVID-19 (Fig. 4B).

The Green module exhibited higher expression in severely ill patients at T1 and T2 but fell to comparable levels to moderate patients at T3 (Fig. 4B). Expression correlated strongly with the neutrophil/lymphocyte ratio, IL-6 and CRP (Fig. 4A). Involving neutrophil and cytokine-associated GOs and genes (*CD177, MCEMP1, FFAR3*), the Green module overlapped with time series clusters 2 and 3.

The Red module overlapped with time series clusters 3 and 4, with antiviral and IFN-associated GO terms considerably overrepresented. In keeping with this, expression of this module correlated inversely with days from symptom onset and fell markedly over time in patients with both severe and moderate disease. There was a modest inverse correlation between the Red module and WHO severity score (Fig. 4A), while expression at given timepoints was comparable in the two patient groups (Fig. 4B).

The Brown module, characterized by RNA processing and ribosome biogenesis GO terms, correlated inversely with disease severity, and expression was stable over time.

The Salmon module consisted of immunoglobulin and other B cell-related genes also present in the immunoglobulin subcluster of time series cluster 4, and expression of the Salmon module similarly seemed to fall at T3 in patients with severe disease, suggesting compromised B cell immunity later in severe disease (Fig. 4B). However, there was no significant correlation between expression of the Salmon module and disease severity at sampling.

### Leukocyte subsets perturbed in severe COVID-19

**Figure 5:**
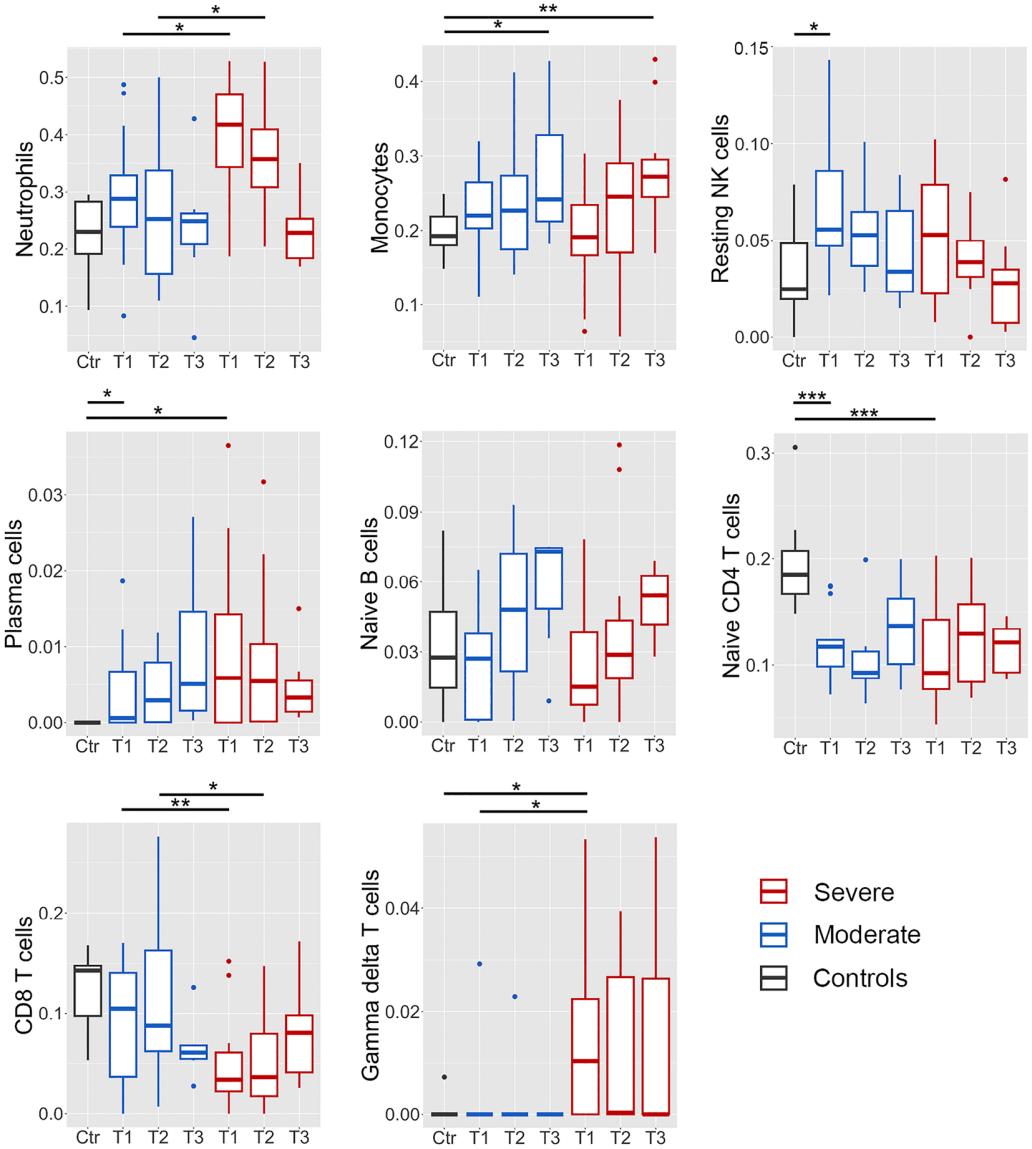
CIBERSORT. Leukocyte differential counts imputed by CIBERSORT, by patient group and time point. Severe in red, moderate in blue. Healthy controls (Ctr) in grey. Median and 1^st^/3^rd^ quartile indicated. *p < 0.05; **p < 0.01; ***p < 0.001.

Finally, we used CIBERSORT to impute differential leukocyte proportions (Fig. 5, Supplementary File 1). CIBERSORT results correlated well with relative differential counts determined by routine clinical methods (Neutrophils: rho 0.80, Monocytes: rho = 0.58, Lymphocytes: rho = 0.79, data in Supplementary File 1).

Neutrophil signatures were significantly elevated in early COVID-19, with the highest proportion in patients with severe disease. In both patient groups, neutrophils were highest at T1, before falling to near-normal levels by T3. There was a consistent, but non-significant trend of increasing monocyte and naïve B cell proportions in COVID-19 from levels similar to healthy controls on at T1, whereas plasma cells were increased at all timepoints in COVID-19 compared to healthy controls with no significant difference between patient groups. Gamma-delta T cells were elevated in severe COVID-19 at T1.

COVID-19 was associated with reduced naïve CD4 T cells compared to healthy controls, with no significant difference between the disease severity groups. At T1 and T2, CD8 T cells were decreased in patients with severe COVID-19, with no difference found between patients with moderate disease and healthy controls. At T3, however, CD8 T cell proportions were reduced to a similar extent in patients with both severe and moderate COVID-19.

## Discussion

This study presents longitudinal whole blood transcriptomic data from a cohort of moderately and severely ill COVID-19 patients and investigates immune mechanisms contributing to severe COVID-19 disease. Our most robust finding was the pronounced activation of neutrophils, platelets and coagulation in COVID-19, with a clear positive association with disease severity. We identified two distinct trajectories of neutrophil-associated genes suggesting functional alterations of neutrophils over the disease course. Interferon signaling and antiviral response genes were enriched in early COVID-19 before diminishing over time. Overall, our analysis suggests a quantitative rather than a qualitative difference in the host response in patients with severe and moderate COVID-19. Two exceptions to this pattern were the observations of modest differences in the trajectories of both IFN- and B cell-associated genes in the two patient groups.

Numerous studies associating an elevated neutrophil/lymphocyte ratio with the need for mechanical ventilation and increased mortality^27,28^ were early evidence of systemic inflammation and dysregulated circulating neutrophils in severe COVID-19. Further studies applying RNA sequencing and high-dimensional cytometry have elaborated on these observations, demonstrating increased frequencies of immature neutrophils and subsets with immunosuppressive properties in severe disease^1,2,4–7,29^. In our microarray analysis of bulk RNA in longitudinal samples, we make concordant observations, identifying two distinct trajectories of neutrophil-associated genes. Time series cluster 2 and the Green WGCNA module, which both contained neutrophil activation markers including *CD177*, exhibited high expression at baseline (T1), particularly in severe patients, before falling over time. CD177 has been associated with severe COVID-19 both at the mRNA and protein level^1,30,31^, but in our data the same trajectory was evident in the proportion of circulating neutrophils by both clinical cytometry (Supplementary Fig. 8) and CIBERSORT (Fig. 5), suggesting that elevated CD177 may merely reflect the relative number of activated neutrophils represented in bulk whole blood RNA.

On the other hand, despite a decrease over time in the proportion of circulating neutrophils, expression of a large number of neutrophil-associated genes in time series cluster 1 and the overlapping MidnightBlue module increased from T1 to T3, suggesting considerable functional alterations in the neutrophil population over the disease course. Furthermore, expression of these gene clusters correlated strongly with WHO disease severity score and with circulating GDF-15, a robust marker of disease severity and poor prognosis^32^. Immature neutrophils expressing many of the genes in these clusters (including *LTF*, *LCN2*, *ELANE*, *MMP8*, *DEFA3/4*, *CEACAM8*, *BPI*, *CD24* and *AZU1*) have been associated with severe disease in studies using both bulk RNA-seq of isolated neutrophils^1,3,4^ and single cell RNA-seq^5,7,29^. While our clinical cytometry data did not include band forms, a study by Morrissey et al. identified a subset of low-density neutrophils expressing *CD24, DEFA3/4, ELANE, and ARG1* which was associated with severe COVID-19 and increased in severe patients over time, confirming that the cells were morphologically consistent with immature neutrophils with band-shaped nuclei^4^. In a study utilizing bulk RNA-seq of purified neutrophils, LaSalle et al. found that immature neutrophil signatures, including clusters characterized by *CEACAM8/CD24* expression, were overrepresented in severe COVID-19 and increased over time^3^. Similarly, Schulte-Schrepping et al. identified immature neutrophil subsets in single cell RNA-seq data which expressed *MPO, DEFA4, BPI, CEACAM8, ARG1 and MMP8*, were associated with severe disease and increased with time^5^. Our data, while less granular, thus lends further support to reports suggesting that severe COVID-19 is accompanied by emergency myelopoiesis^5,6,33^.

*MPO* and *ELANE* are involved in the formation and release of neutrophil extracellular traps (NETs), and their elevated expression in our data is concordant with studies observing both higher circulating concentrations of NET markers in patients with severe COVID-19^3,34–36^ and NETs in the lung vasculature in fatal disease^33,34,37^. Expression of the Magenta module, including several integrin genes, similarly increased over time and was higher in patients with severe disease. Integrins have been shown to facilitate platelet binding to neutrophils and induction of NET formation in vitro^38^, and platelets enriched for integrin genes and the wound healing GO term have been associated with severe COVID-19^39^.

We observed a consistent downregulation of class II HLA genes, which was more pronounced in severe disease. HLA II downregulation on monocytes and dendritic cells has been associated with severe COVID-19 in multiple studies^6,40,41^ and is an established marker of immune dysfunction in sepsis^42^. Compromised antigen presentation may contribute to inefficient T cell responses and the lymphopenia consistently observed in severe COVID-19^43^. Interestingly, while our data also suggested reduced CD8 T cell proportions and impaired T cell function in severe disease, CIBERSORT indicated an increase of γδ T cells in the same group in early disease. This contrasts with findings from other studies, in which the frequency of this HLA-independent T cell subset has been reduced^8,44^ or unchanged^45^ in severe COVID-19.

Moreover, we observed increased expression of *CD274*/PD-L1, which may further contribute to reduced T cell function in severe COVID-19. PD-L1 can be induced by both type I and II IFNs^46,47^, and *CD274/*PD-L1 clustered with other IFN-related genes in our time series analysis. IFN-associated pathways were enriched in COVID-19 at T1 and T2, and gene clusters including interferon-associated genes were most highly expressed at T1 before diminishing over time. This is concordant with several previous studies^3,10,48–50^. However, comparing patients who developed severe vs. moderate disease, IFN-associated gene expression was modestly elevated in the severe group only at T1, before decreasing to a level comparable to moderate patients at T2 and T3. Higher ISG expression in severe patients at T1 could be secondary to higher levels of viral RNA in the early phase of disease. The subsequent decline of ISG expression in this group to the level of moderate patients does not, however, support an important role of IFN signaling in driving the immunopathogenesis of severe COVID-19.

The association between IFNs, ISGs and COVID-19 severity have been somewhat contradictory in previous studies. While Hadjadj et al*.* found low circulating IFN-α and downregulated whole blood ISGs in patients who developed severe COVID-19 vs. patients with mild or moderate disease^11^, Galani et al*.* and Krämer et al*.* measured higher levels of circulating IFN-α in severe patients^10,14^. Interestingly, while Lucas et al.^51^ observed that circulating IFN-α fell in moderately ill patients over time, but rose in severe patients, IFN-α fell over time in severe patients in the Galani, Krämer and Hadjadj studies. Studies by Fava et al. and Lee et al*.* associated increased expression of ISGs in monocytes with severe COVID-19 at admission and later in disease, respectively^13,52^, whereas Krämer et al*.* detected ISG induction in NK cells in severe patients, which diminished over time^14^. Similarly, LaSalle et al*.* observed ISG signatures in neutrophils in early disease which diminished over time^3^, except in fatal cases, where the IFN response persisted. Other studies have associated the abrogation of IFN signaling by either inborn errors or auto-antibodies with critical COVID-19^9,12,52^.

Interpretation of our transcriptomic data vs. protein data from other studies is complicated by the possibility that ISGs in peripheral blood leukocytes are induced by other mechanisms than circulating IFNs*.* Furthermore, circulating transcripts are not necessarily representative of ongoing immune processes in the lungs. Arunachalam et al. observed strong induction of ISGs in circulating leukocytes correlating with concentrations of circulating type I IFNs, but very low expression of the corresponding IFN transcripts^48^. While we detected robust severity-associated differences in circulating cytokines such as IL-6 and GDF-15 (Supplementary Fig. 8), the corresponding transcripts were low or undetectable in our whole blood data. Hadjadj et al. made a similar observation of low levels of IL-6 transcripts concurrently with high circulating concentrations of IL-6^11^.

Many cross-sectional studies have demonstrated an expansion of circulating plasmablasts in COVID-19^53–55^. While we made the same observation, our data also suggest a reduction of immunoglobulin gene expression and a diminished CIBERSORT plasma cell signal in severe patients at T3. As there was no difference in circulating SARS-CoV-2-specific antibody levels between severe and moderate patients, our finding could potentially reflect a reduction in polyclonal B cell activation due to immune exhaustion in severe disease, or sequestering of plasma cells in the lungs^56^.

Finally, our analysis suggested reduced expression of protein synthesis-related genes in COVID-19 compared to healthy controls. While the differences in blood cell composition indicated by CIBERSORT could play a role in this, downregulation of ribosome and translation GOs in severe COVID-19 has previously been demonstrated in both bulk leukocyte RNA^57^ and circulating monocytes^52^*.*

A strength of this study was the serial sampling at up to three timepoints, providing insights into the kinetics of the immune response in patients hospitalized with COVID-19 in the absence of vaccine protection. On the other hand, the study was limited by a modest sample size, which along with the inherent variability of human gene expression data contributed to the paucity of differentially expressed genes between patients with severe and moderate disease. Furthermore, while we strived to include and sample patients at comparable timepoints from symptom onset, uncertainty regarding self-reported symptom onset and logistical challenges hampering uniform intervals between sequential samples may have reduced the sensitivity of our analysis to detect severity-related differences and precisely characterize time courses. However, our key findings were validated by two complementary clustering approaches: selecting genes by repeated differential expression analyses and applying PART clustering, our published time series analysis^25^ is suitable for identifying smaller genomic clusters, whereas WGCNA is more likely to detect larger pathways. Finally, our analysis of bulk RNA has inherent limitations with regard to ascribing observed gene expression patterns to specific cellular subsets and/or tissues, but our main conclusions are concordant with studies analyzing single cell data^5–7,39,40,52^.

Inclusion and sampling for this study was performed during the first wave of COVID-19 in Norway, and thus generalization of our findings to disease caused by currently circulating variants of SARS-CoV-2 and disease in vaccinated individuals should be made with caution. However, an advantage of this early sampling is that our data is unaffected by immunomodulatory treatment which has later become standard of care.

In conclusion, this study provides further data on the dynamics of the systemic immune response in SARS-CoV-2 infection requiring hospitalization, and characteristics of severe disease. In particular, our data reinforce the notion that the development of moderate and severe COVID-19 is accompanied by a shift to a more immature circulating neutrophil phenotype, which is more pronounced in severe disease. On the other hand, we observed falling IFN signaling over time, with minor differences between severe and moderate disease. While antiviral responses in the days following infection likely play an important role in determining COVID-19 severity, our data do not support an important role for ongoing IFN signaling in the hyperinflammatory phase of disease. However, decreased expression of class II HLAs and increased expression of the IFN-stimulated PD-L1 may contribute to compromised T and B cell function in COVID-19.

### Supplementary Information

Supplementary Information 1.

Supplementary Information 2.

Supplementary Information 3.

Supplementary Information 4.

Supplementary Information 5.

Supplementary Information 6.

Supplementary Information 7.

Supplementary Information 8.

## Data Availability

Microarray data is available at the Gene Expression Omnibus, with accession number GSE213313. (Link: https://www.ncbi.nlm.nih.gov/geo/query/acc.cgi?acc=GSE213313). R code to perform the analysis is available at: https://github.com/Ylefol/CovMech.
