# Author Correction: Longitudinal whole blood transcriptomic analysis characterizes neutrophil activation and interferon signaling in moderate and severe COVID-19

**DOI:** 10.1038/s41598-023-39872-2

**Published:** 2023-08-04

**Authors:** Christian Prebensen, Yohan Lefol, Peder L. Myhre, Torben Lüders, Christine Jonassen, Anita Blomfeldt, Torbjørn Omland, Hilde Nilsen, Jan‑Erik Berdal

**Affiliations:** 1 Department of Infectious Diseases, Oslo University Hospital, Kirkeveien 166, 0450 Oslo, Norway; 2 Institute of Clinical Medicine, University of Oslo, Oslo, Norway; 3 Department of Microbiology, University of Oslo, Oslo, Norway; 4 Department of Cardiology, Akershus University Hospital, Lørenskog, Norway; 5 Department of Clinical Molecular Biology, Akershus University Hospital, Lørenskog, Norway; 6 Center for Laboratory Medicine, Østfold Hospital Trust, Grålum, Norway; 7 Department of Microbiology and Infection Control, Akershus University Hospital, Lørenskog, Norway; 8 Department of Infectious Diseases, Akershus University Hospital, Lørenskog, Norway

Correction to: *Scientific Reports* 10.1038/s41598-023-37606-y, published online 26 June 2023

The original version of this Article contained an error in one of the author names. Peder L. Myhre was incorrectly given as Peder Myhre.

The original Article has been corrected.

