# Supplementary material for: Longitudinal whole blood transcriptomic analysis characterizes neutrophil activation and interferon signaling in moderate and severe COVID-19
Source: Sci Rep. 2023 Jun 26;13:10368. doi: 10.1038/s41598-023-37606-y (PMC10293211; doi:10.1038/s41598-023-37606-y)
Supplement: Supplementary file 8 — Supplementary Information 8. [file 41598_2023_37606_MOESM8_ESM.pdf]

Supplementary Figures

Title: Longitudinal whole blood transcriptomic analysis characterizes neutrophil activation and interferon signaling in moderate and severe COVID-19.

Authors:
Christian Prebensen (co-first, corresponding)
Yohan Lefol (co-first)
Peder Myhre
Torben Lüders
Christine Jonassen
Anita Blomfeldt
Torbjørn Omland
Hilde Nilsen
Jan-Erik Berdal

Supplementary Figure 1:

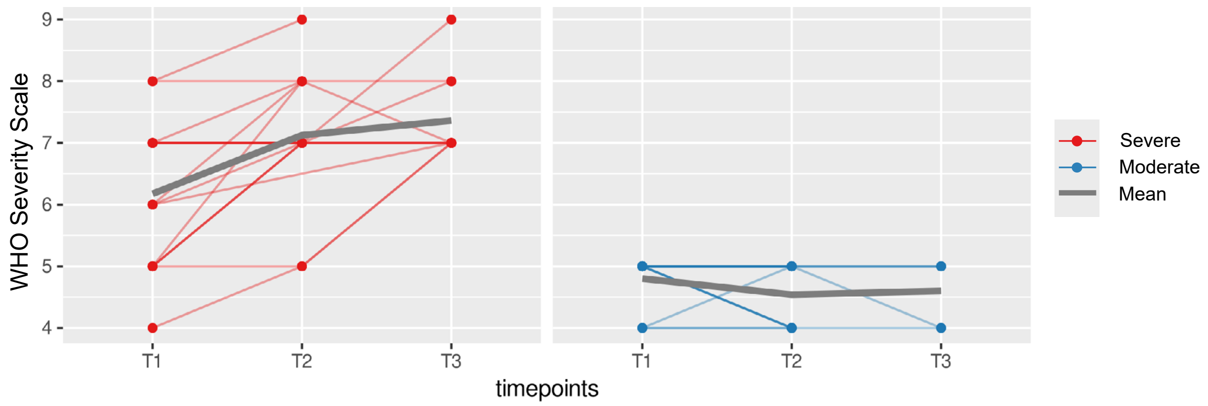

Clinical trajectories. WHO severity scale over time in severe (red) and moderate (blue) patients. Mean values in grey.

Supplementary Figure 2:

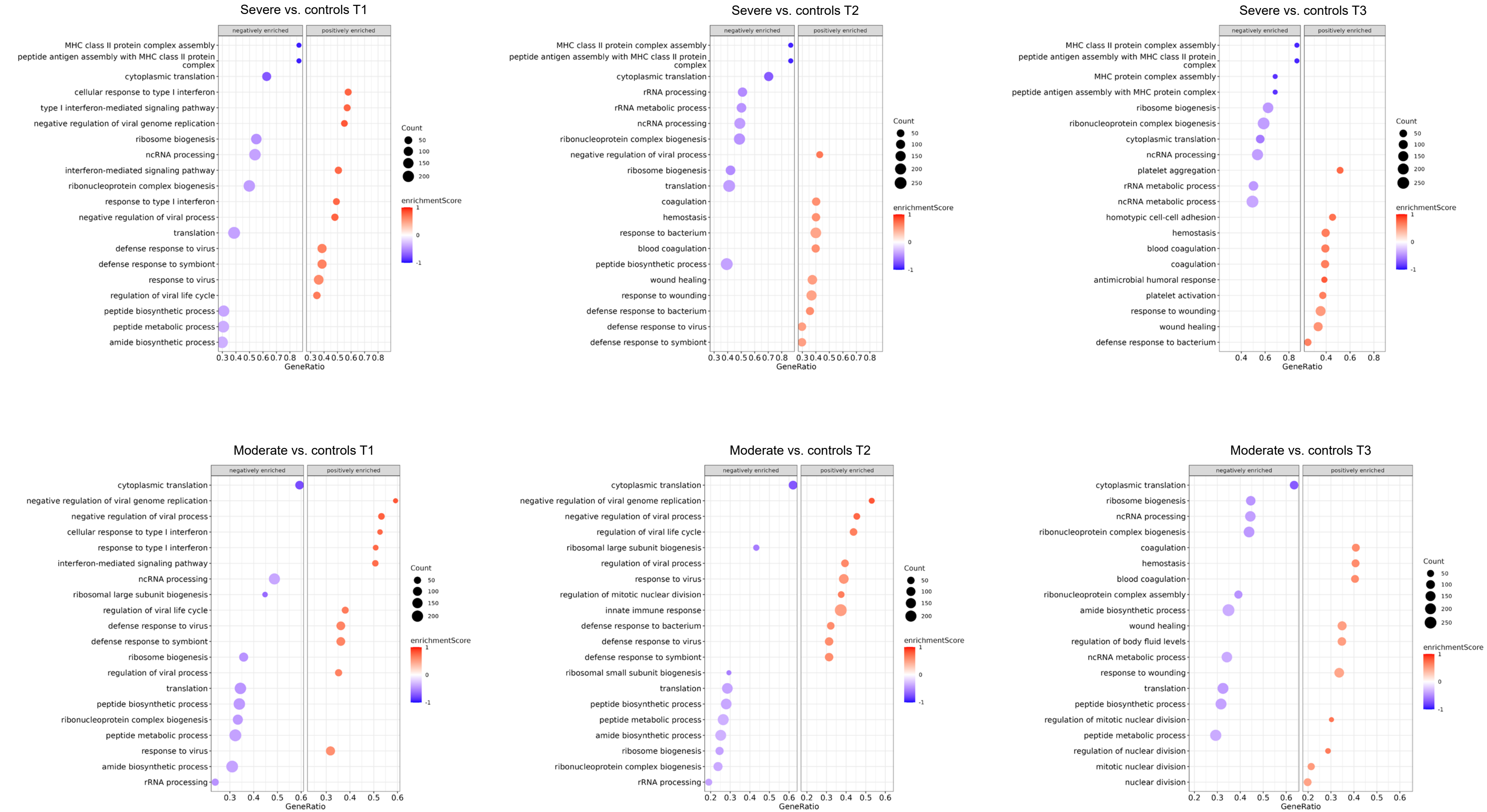

Gene set enrichment analysis (GSEA) in severe and moderate patients vs. healthy controls. Top 20 GO terms for each comparison indicated.

Supplementary Figure 3:

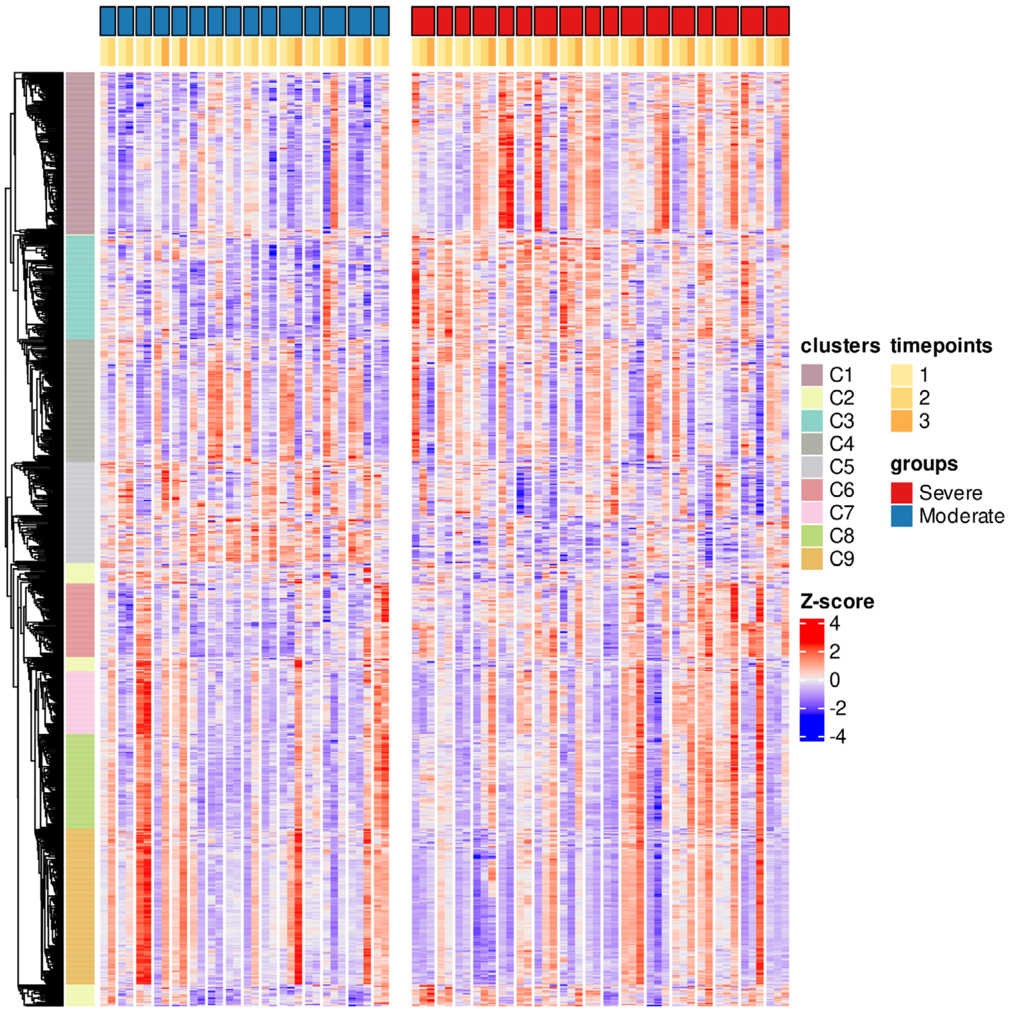

Time Series heatmap. Heat map showing the results of PART clustering in the time series analysis.

Supplementary Figure 4:

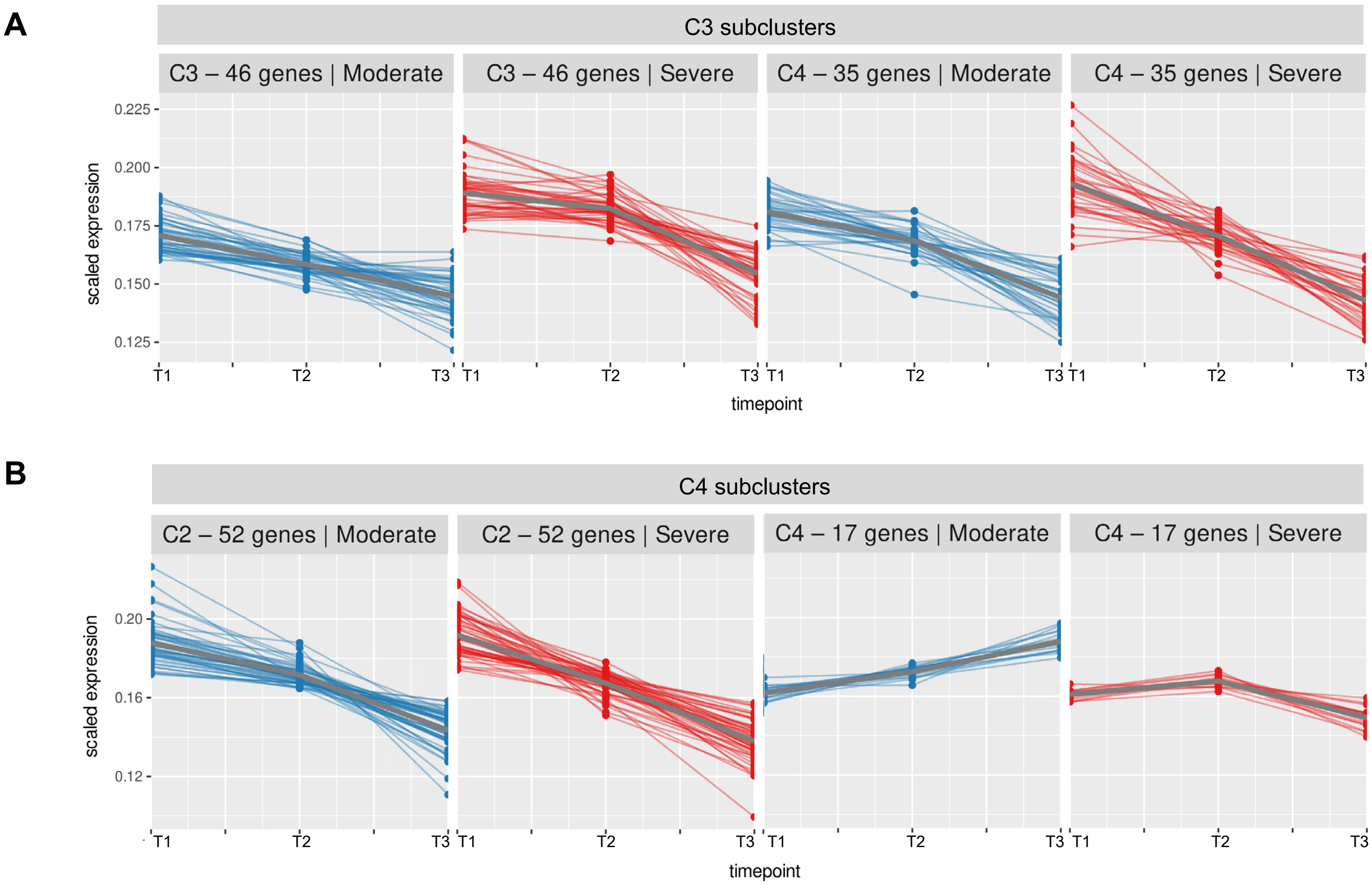

Time series subclusters. Trajectories of subclusters in A: C3 and B: C4. Patients with severe disease in red, moderate disease in blue. Mean scaled expression in grey.

Supplementary Figure 5:

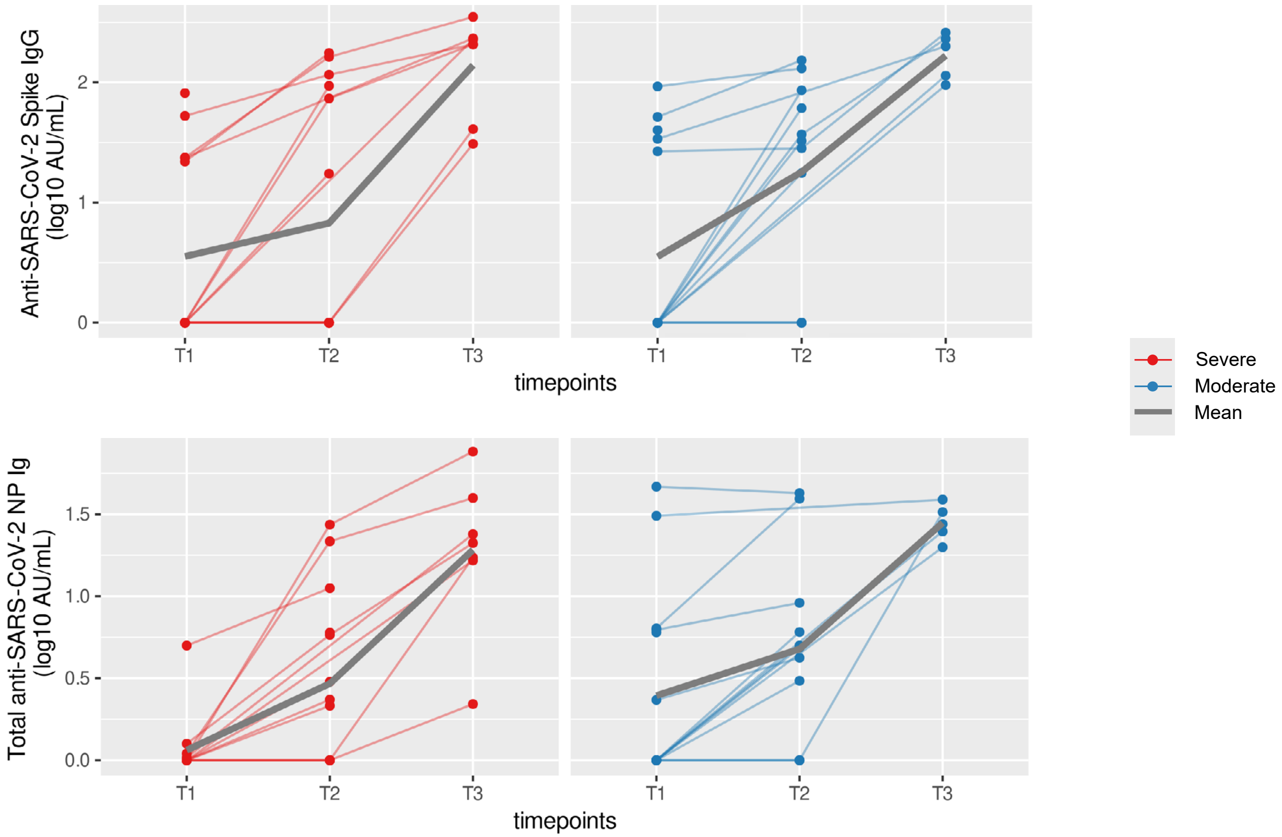

SARS-CoV-2-specific antibody levels. Levels of SARS-CoV-2-specific antibodies in serum samples, per patient, timepoint and severity group. Mean value per group in grey.

Supplementary Figure 6:

A

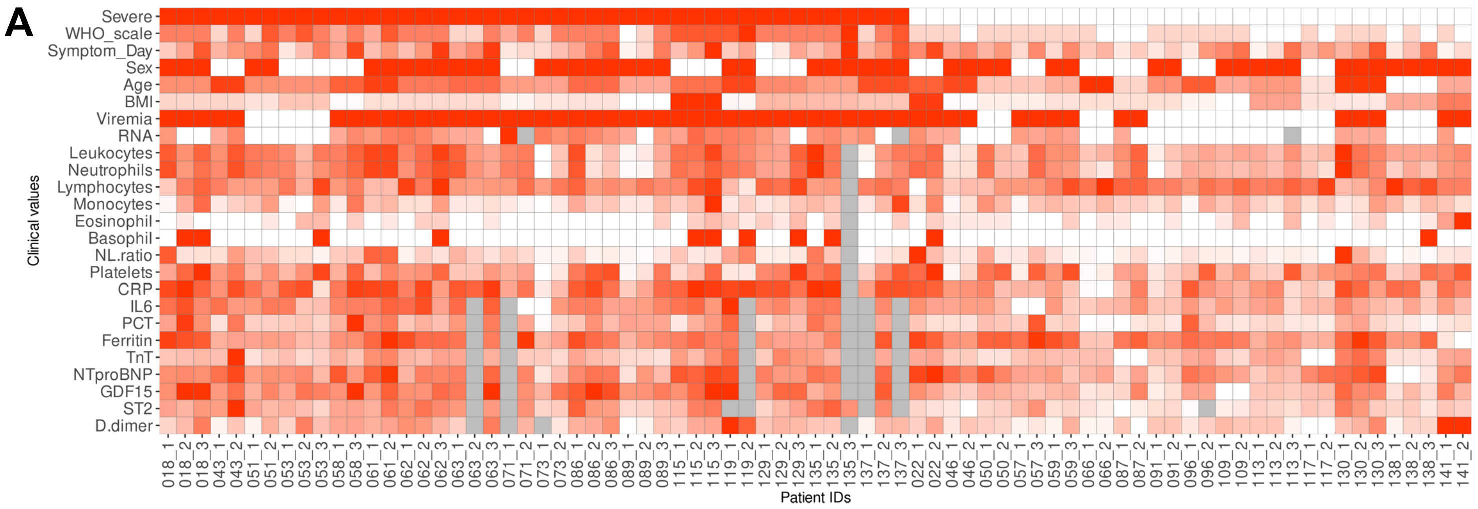

Clinical trait heatmap. Heat map of clinical traits and biomarkers for each sample. Biomarker variables are log-transformed. Grey boxes indicate missing data.

B

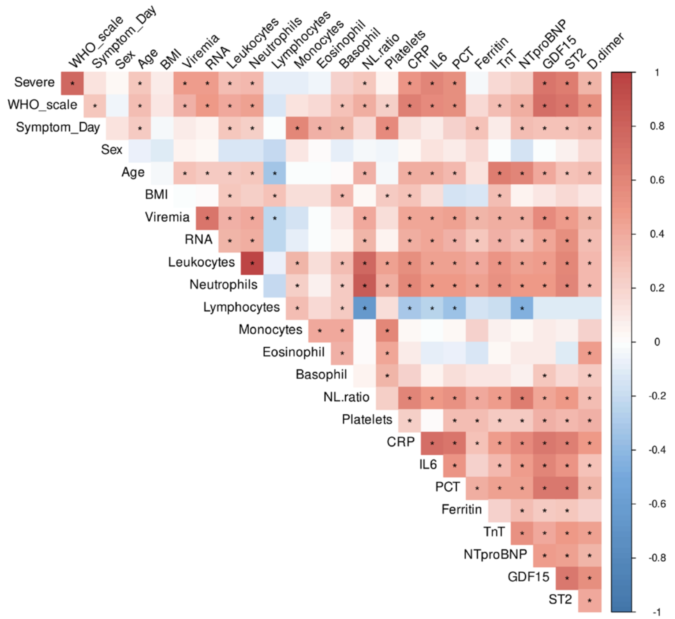

Correlation between clinical traits and biomarkers. Color indicates the correlation statistic, scale on the right. Dots indicate a p-value < 0.05.

Supplementary Figure 7:

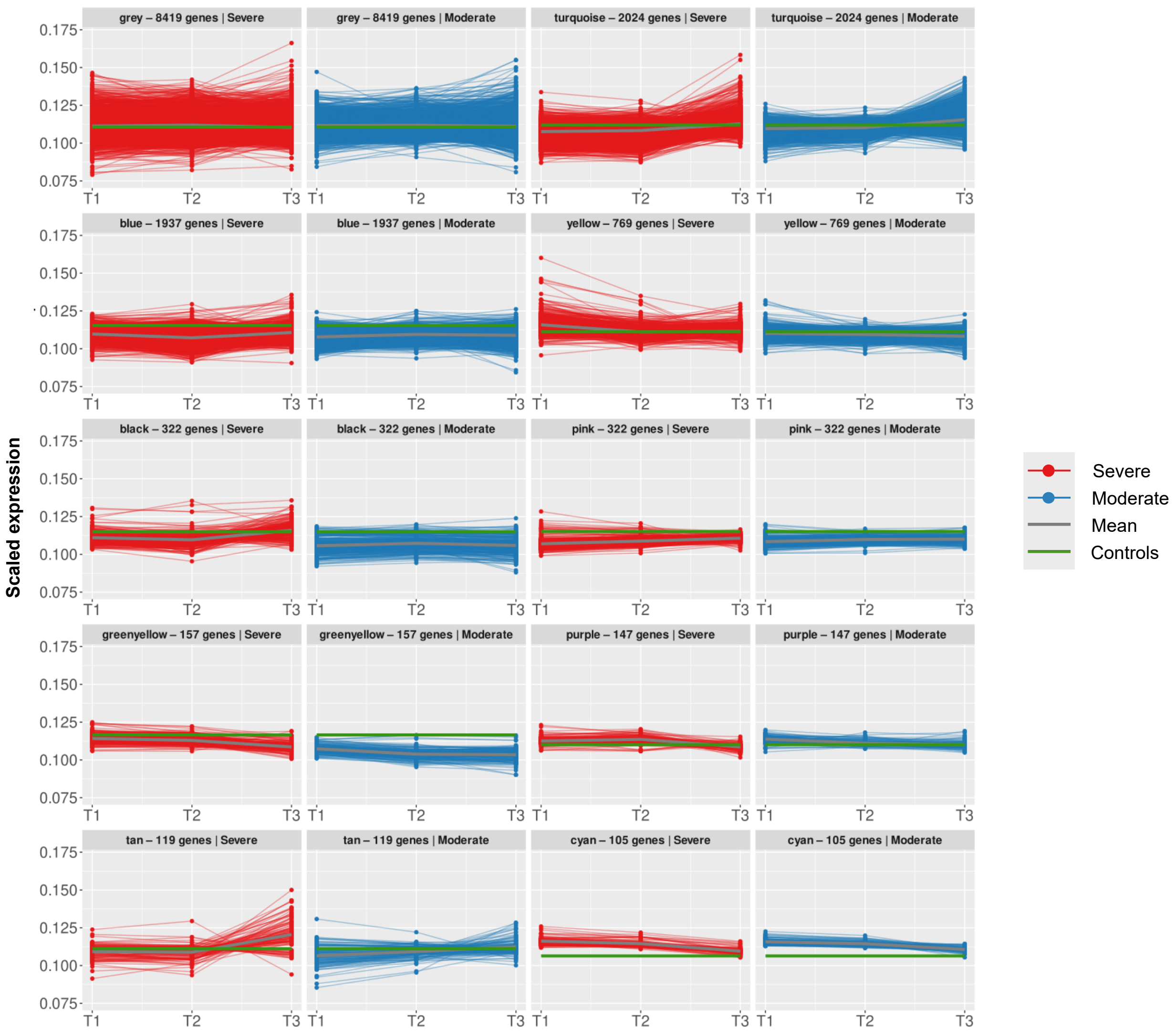

Remaining WGCNA module trajectories. Gene trajectories over time in severe (red) and moderate (blue) patients. Mean scaled expression in patients and healthy controls in grey and green, respectively.

Supplementary figure 8:

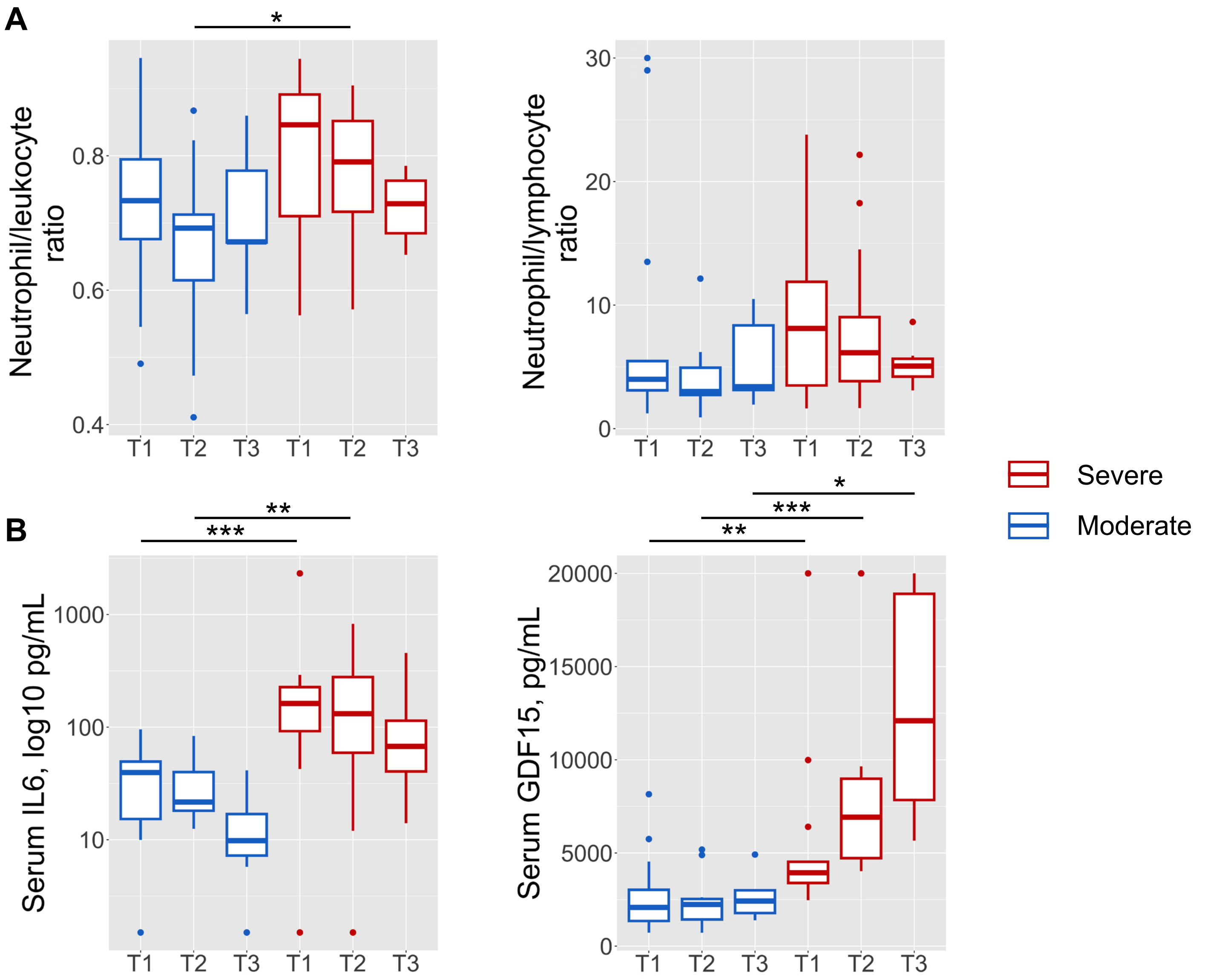

Biomarkers: A: Leukocyte ratios by clinical cytometry and B: serum concentrations of cytokines, per severity group and timepoint. Moderate in blue, severe in red. *: p<0.05 **: p<0.01 ***: p<0.001
